# A meta-analysis on the prevalence and characteristics of severe malaria in patients with *Plasmodium* spp. and HIV co-infection

**DOI:** 10.1038/s41598-021-95591-6

**Published:** 2021-08-17

**Authors:** Aongart Mahittikorn, Kwuntida Uthaisar Kotepui, Giovanni De Jesus Milanez, Frederick Ramirez Masangkay, Manas Kotepui

**Affiliations:** 1 Department of Protozoology, Faculty of Tropical Medicine, Mahidol University, Bangkok, Thailand; 2 Medical Technology, School of Allied Health Sciences, Walailak University, Tha Sala, Nakhon Si Thammarat, Thailand; 3 Department of Medical Technology, Faculty of Pharmacy, University of Santo Tomas, Manila, Philippines; 4 Department of Medical Technology, Institute of Arts and Sciences, Far Eastern University-Manila, Manila, Philippines

**Keywords:** Malaria, Parasitic infection

## Abstract

Co-infection with malaria and human immunodeficiency virus (HIV) increases the severity and mortality rates of both diseases. A better understanding of the effects of co-infections could help in the diagnosis, prompt treatment, prevention, and control of malarial parasites among HIV-infected patients. In this systematic review and meta-analysis, we estimated the prevalence and characteristics of severe malaria (SM) caused by co-infection with HIV. We included relevant studies that were conducted between the years 1991 and 2018 and reporting on SM. We pooled the prevalence of SM in patients with co-infection, pooled odds ratios of SM in patients with co-infection and *Plasmodium* mono-infection, and differences in laboratory parameters such as parasite density and leucocyte counts, between co-infected and *Plasmodium* mono-infected patients. The meta-analysis included 29 studies (1126 SM cases). The pooled prevalence of SM in co-infected patients using the data of 23 studies (SM = 795 cases, all co-infection cases = 2534 cases) was 43.0% (95% confidence interval [CI] 31.0–56.0%; I^2^, 98.0%). Overall, the odds of SM from 18 studies were pooled. The odds of SM were significantly higher in co-infected patients than in *Plasmodium* mono-infected patients (OR 2.41; 95% CI 1.43–4.08; I^2^ = 85%; *P* = 0.001) and also significantly higher in children (OR 9.69; 95% CI 5.14–18.3; I^2^, 0%; *P* < 0.0001; two studies) than in adults (OR 2.68; 95% CI 1.52–4.73; I^2^, 79.0%; *P* = 0.0007; 12 studies). Co-infected patients with SM had a higher parasite density than those with *Plasmodium* mono-infection when the data of seven studies were analysed (SMD, 1.25; 95% CI 0.14–2.36; I^2^, 98.0%; *P* = 0.03) and higher leukocyte counts when the data of four studies were analysed (MD, 1570 cells/µL; 95% CI 850–2300 cells/µL; I^2^, 21.0%; *P* < 0.0001). Thus, the prevalence of SM among patients co-infected with *Plasmodium* spp. and HIV is high. Because co-infections could lead to SM, patients with *Plasmodium* spp. and HIV co-infection should be identified and treated to reduce the prevalence of SM and the number of deaths.

## Introduction

Malaria remains one of the most dangerous diseases affecting the world’s population with about 228 million cases and 405,000 deaths from malaria globally^1^; most of the malaria cases (93%) and deaths (94%) were found in the African Region^1^. In areas with stable malaria, human immunodeficiency virus infection (HIV) and acquired immune deficiency syndrome (AIDS) increase the risk of malaria infection, especially in adults with advanced immunosuppression^2,3^. HIV infection remains a major health problem with approximately 37.9 million people living with HIV and 770,000 deaths observed at the end of 2018^4^.

Severe malaria (SM) is defined by the World Health Organization 2014 by the presence of malaria parasites in the blood of patients with potentially fatal manifestations, including impaired consciousness, acidosis, hypoglycaemia, severe malarial anaemia (SMA), renal impairment, jaundice, pulmonary oedema, significant bleeding, shock, and hyperparasitaemia^5^. In Africa, many children develop three overlapping syndromes—cerebral malaria, severe malarial anaemia, and respiratory distress—and the prognoses and ages at presentation differ^6^. The sequestration of infected red blood cells (RBCs) in the microvascular system of patients with *Plasmodium falciparum* infections is the main factor of severe malaria^7^. SM can be caused not only by *P. falciparum* but also by *Plasmodium knowlesi*^8^, *Plasmodium vivax*^9,10^, *Plasmodium malariae*^11^, and *Plasmodium ovale*^12^, although in fewer people. However, the mechanism remains poorly understood.

Co-infection with *Plasmodium* spp. and HIV is likely to occur because of the high prevalence of both infections in the same areas, particularly in Sub-Saharan African regions. Data suggest that *Plasmodium* spp. and HIV co-infection result in adverse outcomes particularly in pregnant women and their infants^13^. Previous studies demonstrated that adults infected with HIV were at increased risk of developing severe malaria^14–16^. Moreover, almost all patients with *Plasmodium* spp. and HIV co-infection develop anaemia^17^. A previous meta-analysis of 23 studies demonstrated that the development of anaemia increased by 49% in co-infected pregnant women compared with those who had HIV in mono-infection^18^. In addition, mono-infection with either malaria or HIV was associated with haematological alterations, such as anaemia, leukopenia, leucocytosis, thrombocytopenia, monocytosis, and eosinophilia^19,20^. However, there is limited information on the impact of *Plasmodium* spp. and HIV co-infection on SM and a better understanding of the impact of co-infections could help in the diagnosis, prompt treatment, prevention, and control of malaria parasites among HIV-infected patients. Thus, the primary aim of our study was to generate a pooled prevalence estimate of SM among patients co-infected with *Plasmodium* spp. and HIV. Our secondary aim was to compare the odds of SM caused by *Plasmodium* spp. and HIV co-infections with those of SM caused by *Plasmodium* mono-infection. The third aim was to identify the differences in laboratory parameters between patients with *Plasmodium* spp. and HIV co-infection and those with *Plasmodium* mono-infection.

## Results

### Study selection

**Figure 1:**
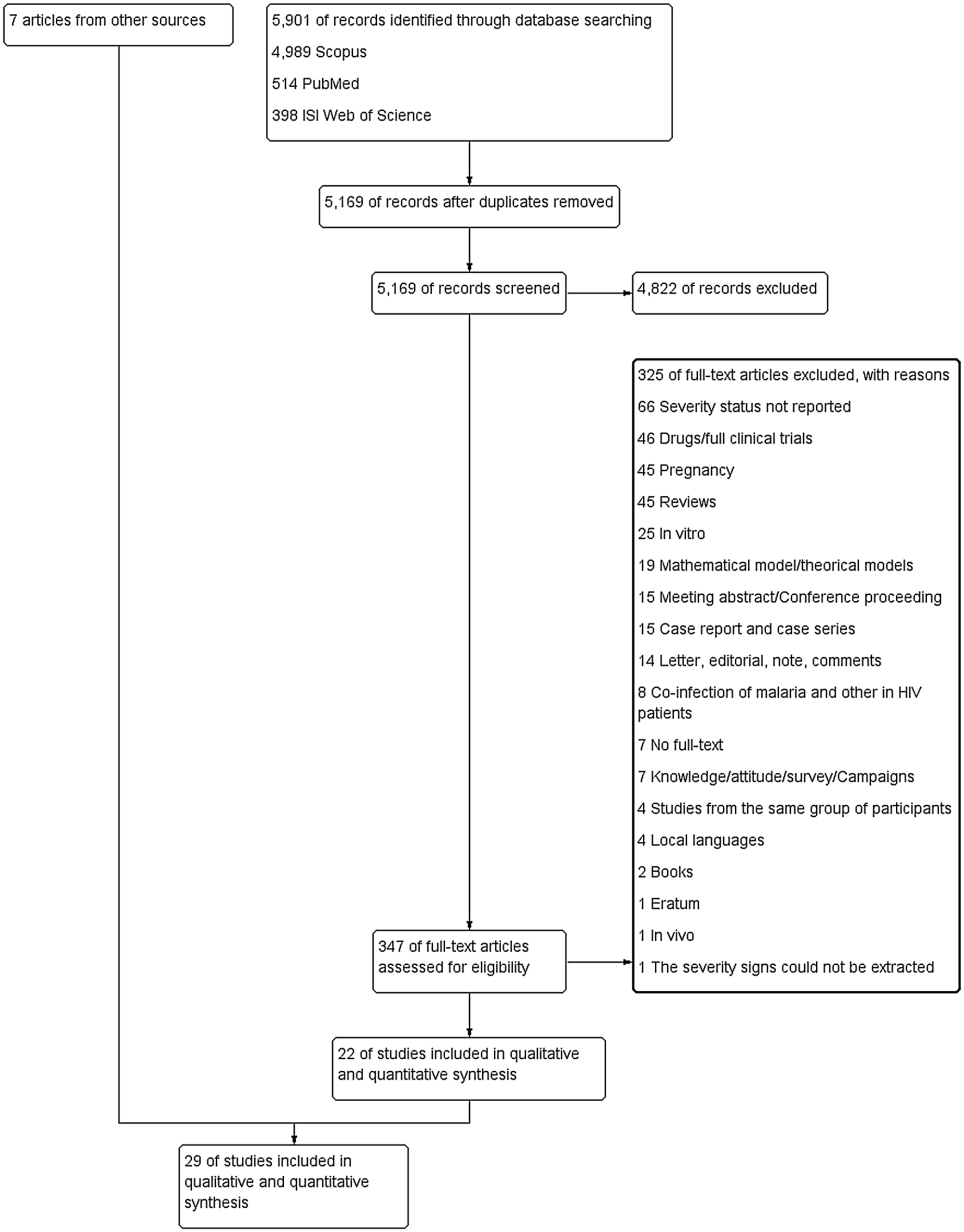
PRISMA diagram. Flowchart for study selection.

A total of 5901 articles were identified by the initial search. After removing duplicates, 5169 articles remained for further consideration. Article titles and abstracts were screened, leading to the exclusion of additional 4822 articles. Further assessments of 347 full-text articles were performed, and 22 of these met the inclusion criteria^2,21–41^, whereas 325 were excluded (Fig. 1). After reviewing the reference list of eligible articles and additional searches, seven additional articles^15,17,42–46^ were included in the present study. Eventually, a total of 29 studies were included in the systematic review and meta-analysis.

### Study characteristics

Data from 2534 patients with *Plasmodium* spp. and HIV co-infections across the 23 included studies^2,15,17,21–26,28–30,32,33,36–43,46^ (range 9–1071) and six studies^27,31,34,35,44,45^ reporting SM in patients with *Plasmodium* spp. and HIV co-infection were analysed in the present study (Table 1). Of the 29 included studies, 28 (96.6%) were conducted in African countries: 6 (20.7%) in Malawi^27,31,34,35,37,44^; 5 (17.2%) in Mozambique^2,23,24,30,40^; 3 (10.30%) in Kenya^25,29,46^; 2 (6.90%) each in Ghana^17,41^, Cameroon^38,39^, South Africa^15,42^ and Ethiopia^21,26^ and 1 (3.40%) each in Nigeria^22^, Zambia^28^, Gabon^32^, Uganda^43^, Congo^33^ and Burundi^45^. The other study involved patients in France^36^. Most of the included studies were cross-sectional studies (20/29, 69%)^2,17,21–27,29,30,33,35,37–42,45^, whereas two were prospective cohort studies^15,46^, four were case–control studies^28,31,34,43^, and one was retrospective study^36^. Most of the studies had included adults aged > 15 years^2,15,21,23,24,28,32,37,38,40–42,45^, children aged < 15 years (10/29, 34.5%)^22,25,27,29,31,34,35,43,44,46^ and any age group^17,26,30,33,36,39^, respectively. Most participants at enrolment reflected patients with malaria (16/29, 55.2%)^15,21,24,25,27–31,33–37,43,45^, patients with HIV/AIDS^17,22,26,32,39,41^, with undefined^23,38,40,46^ febrile^2,42^ and other conditions/diseases^44^. The most common diagnostic method for the detection of *Plasmodium* spp. among the included studies was microscopy (25/29, 86.2%), whereas the most common method for the identification of HIV was polymerase chain reaction (12/29, 41.4%). *P. falciparum* was the only *Plasmodium* spp. reported among HIV-positive patients^2,15,21–25,27–32,34–38,40–46^; three reports did not specify the *Plasmodium* spp.^17,33,39^ and one study^26^ focused on mixed infection with *P. falciparum* and *P. vivax* among HIV-positive patients but did not specify the exact *Plasmodium* spp. among patients with SM. Table 2 lists the laboratory data on parasite density, leukocyte counts and differential counts of *Plasmodium* spp. and HIV co-infected patients with SM *versus Plasmodium* spp. mono-infected patients with SM.

**Table 1:** Characteristics of the included studies.

No.	Author, year	Study area (years of the survey)	Study design	Age range	Gender	Participants	HIV status	*Plasmodium* mono–infections	Detection method for *Plasmodium* sp.	Severe malaria (mono-infections)	HIV mono-infections	Detection method for HIV	Co-infections
1.	Addissie et al. (2007)^21^	Ethiopia (2003–2004)	Cross-sectional study	15–34	Male (203), Female (104)	337 *P. falciparum* Severe 62 Prostration 18 Hyperparasitemia 17 Cerebral manifestations 16 Other 18	Recently diagnosed	323 (*P. falciparum*)	Microscopy	Severe malaria 52 Cerebral malaria 16, prostration 18, hyperparasitemia, and other complications (estimated 18)	NA	ELISA	14 (*P. falciparum*) Severe malaria 3 (*P. falciparum*)
2.	Amodu-Sanni et al. (2020)^22^	Nigeria (2016)	Cross-sectional study	3 months to 15 years	Male (143), Female (137)	140 HIV positive, 140 HIV negative	Undertreated	132 (*P. falciparum*)	Microscopy	Severe malaria 84	40	ELISA, PCR	100 (*P. falciparum*) Severe malaria 46 (*P. falciparum*)
3.	Berg et al. (2014)^2^	Mozambique (2011–2012)	Cross-sectional study	18–84	Male (142), Female (126)	212 adults with fever and/or suspected malaria, 56 healthy controls	Recently diagnosed	61 (*P. falciparum*)	Microscopy, RDT, PCR	Severe malaria 24/52 Hypotension (1/52), Respiratory distress (3/52), Hyperpyrexia (6/50), GCS < 11 and/or convulsions (5/61), Bleeding disturbances and/or hemolysis (1/61), Jaundice (3/61) Severe anemia (3/55), Hypoglycaemia (0/47), Renal failure (3/46), Hyperparasitaemia (16/53)	NA	RDT, PCR	70 (*P. falciparum*) Severe malaria 55/66 (*P. falciparum*) Hypotension (0/63), Respiratory distress (15/61), Hyperpyrexia (3/54), GCS < 11 and/or convulsions (6/70), Bleeding disturbances and/or hemolysis (9/70), Jaundice and/or se-bilirubin (13/70), Severe anemia (10/67), Hypoglecemia (5/62), Renal failure (15/63), Hyperparasitemia (33/64)
4.	Berg et al. (2008)^23^	Mozambique (2006)	Cross-sectional study	16–92	Male (167), Female (166)	333 adult patients	Immunosuppressed	8 (*P. falciparum*)	Microscopy	Severe malaria 4 Jaundice 1, Renal failure 2, fatal 1	NA	RDT	12 (*P. falciparum*) Severe malaria 9 (*P. falciparum*) Jaundice 2, Renal failure 6, Fatal 1
5.	Berg et al. (2020)^24^	Mozambique (2011–2012)	Cross-sectional study	≥ 18 years	Male (71), Female (60)	131 *P. falciparum*	Undertreated	61 (*P. falciparum*)	Microscopy, RDT	Severe malaria 28 Bleeding disturbances and/or haemolysis 1, Fatal 1	NA	PCR	70 (*P. falciparum*) Severe malaria 57 (*P. falciparum*) Bleeding disturbances and/or hemolysis 9, Fetal 9
6.	Berkley et al. (2009)^25^	Kenya (1998–2002)	Cross-sectional study	Children aged ≥ 60 days	NA	3,068 severe *P. falciparum*, 592 healthy control	Recently diagnosed	*P. falciparum*	Microscopy	Severe malaria 938/1071	119/684 10/592	ELISA	Severe malaria 133/1071 (*P. falciparum*)
7.	Beyene et al. (2017)^26^	Ethiopia (2012–2013)	Cross-sectional study	< 27 years (139), 27.00–31.99 years (141), 32.0–39.74 years (113), ≥ 39.75 (131)	Male (250), Female (284)	528 people living with HIV/AIDS	Recently diagnosed	NA	Microscopy, RDT	NA	436 Severe anemia 12	NA	92 (52 *P. falciparum*, 37 *P. vivax,* and 3 mixed infections) Severe anemia 25 (*Plasmodium* spp.)
8.	Bronzan et al. (2007)^27^	Malawi (1996–2005)	Cross-sectional study	≥ 6 months old	Male (250), Female (541)	1388 severe malaria	Recently diagnosed	941 severe malaria (*P. falciparum*)	NA	941 severe malaria Cerebral malaria 541/627 Cerebral malaria and severe anemia 291/355, severe anemia 109/137		RDT	Severe malaria 178 (*P. falciparum*) Cerebral malaria 86/627 Cerebral malaria and severe anemia 64/355, severe anemia 28/137
9.	Chalwe et al. (2009)^28^	Zambia (2004–2005)	Case–control study	15–49 years 15–19, 20–29, 30–39, 40–49	Male (42), Female (45)	29 severe malaria, 29 uncomplicated malaria, 29 asymptomatic community controls	Immunosuppressed	32 (*P. falciparum*)	Microscopy, RDT	Severe 2 Jaundice 1	NA	RDT, ELISA, Western blot	55 (*P. falciparum*) Severe malaria 27 (*P. falciparum*) Impaired consciousness 15, Severe anemia 5, Convulsions 6, Jaundice 3, Hypoglycemia 11, Hyperparasitemia 6
10.	Cohen et al. (2005)^15^	South Africa (2001–2002)	A prospective cohort study	15–49 years: Malaria-mono-infection: 29 (17–49) Co-infection: 30 (17–49)	Malaria-mono-infection: Male (178), Female (48) Co-infection: Male (85), Female (25)	502 *P. falciparum* patients	Immunosuppressed	226 (*P. falciparum*)	Microscopy, RDT	Severe malaria 14: Cerebral malaria 4, Severe anemia 0 Renal impairment 9, Shock 2, Acidosis 3, Hypoglycemia 0, Hepatic dysfunction 8	NA	RDT, ELISA	110 (*P. falciparum*) Severe malaria 18 (*P. falciparum*): Cerebral malaria 3 Severe anemia 3, Renal impairment 14, Shock 2, Acidosis 11, Hypoglycemia 1, Hepatic dysfunction 6
11.	Davenport et al. (2010)^29^	Kenya (2004–2006)	Cross-sectional study	3–36 months	Malaria-mono-infection: Male (219), Female (187) Co-infection: Male (14), Female (10)	542 with *P. falciparum*	Undertreated	406 (*P. falciparum*)	Microscopy	Severe anemia 44	NA	RDT, PCR	24 (*P. falciparum*) Severe anemia 14 (*P. falciparum*)
12.	Grimwade et al. (2004)^42^	South Africa (2000)	Cross-sectional study	Adults (> 14 y): Malaria-mono-infection: 28 (18–43) Co-infection: 30 (22–42)	Malaria-mono-infection: Male (211), Female (222) Co-infection: Male (70), Female (110)	1,109 febrile adults	Recently diagnosed	433 (*P. falciparum*)	Microscopy, RDT	Severe malaria 62: Severe malaria 18: Impaired renal function 32, Coma 16, Severe anemia 22, Pulmonary edema 2, Bleeding 0, Acidosis 13, Confusion 18, Jaundice 3	152	ELISA	180 (*P. falciparum*) Severe malaria 18 (*P. falciparum*): Renal failure 28, Coma 16, Severe anemia 14, Pulmonary edema 4, Bleeding 2, Acidosis 15, Confusion 7, Jaundice 9
13.	Hendriksen et al. (2012)^30^	Mozambique (2005–2010)	Cross-sectional study	Malaria-mono-infection: 2.5–23 Co-infection: 3–38 Age group < 15 and > 15 years	Malaria-mono-infection: Male (6314), Female (286) Co-infection: Male (68), Female (55)	896 with suspected severe malaria	Recently diagnosed	600 (*P. falciparum*)	Microscopy, RDT	Severe malaria 549 Coma 454, Convulsions 516, Prostration 137, Shock 21, Severe respiratory distress 38, Severe acidosis 109, Hypoglycemia 33, Severe anemia with respiratory distress 67, Black water fever 28, Severe jaundice 17, Hyperparasitemia 109	NA	RDT, PCR	123 (*P. falciparum*) Severe malaria 107 (*P. falciparum*) Coma 89, Convulsions 74, Prostration 31, Shock 6, Severe respiratory distress 20, Severe acidosis 38, Hypoglycemia 10, Severe anemia with respiratory distress 18, Black water fever 15, Severe jaundice 9, Hyperparasitemia 33
14.	Hochman et al. (2015)^31^	Malawi (1996–2010)	Case–control study	6 months to 12 years	Malaria-mono-infection: Male (39), Female (18) Co-infection: Male (7), Female (8)	103 autopsy tissues	Recently diagnosed	57 (*P. falciparum*)	ELISA	Cerebral malaria 57	NA	IHC	Cerebral malaria 15 (*P. falciparum*)
15.	Huson et al. (2015)^32^	Gabon (2012–2013)	Prospective observational study	age ≥ 18 years	Malaria-mono-infection: Male (44), Female (69) Co-infection: Male (0), Female (14)	103 patients with sepsis 127 patients with malaria, 60 HIV infected control	Undertreated	133 (*P. falciparum*)	Microscopy	0 Fetal 0		RDT, PCR	14 (*P. falciparum*) Fetal 1 (*P. falciparum*)
16.	Imani et al. (2011)^43^	Uganda (2006–2007)	Case–control study	< 12 years	Cerebral malaria: Male (70), Female (30), uncomplicated malaria: Male (92), Female (40), non-malaria: Male (84), Female (36)	352 children: 100 cerebral malaria, 132 uncomplicated malaria, 120 non-malaria	Recently diagnosed	220 (*P. falciparum*)	Microscopy	220 Cerebral malaria 91/220	NA	RDT	12 (*P. falciparum*) Cerebral malaria 9/12 (*P. falciparum*)
17.	Jacques et al. (2019)^33^	Congo (2017–2018)	Cross-sectional study	12–60 years Co-infection 12–60 years	Male (111), Female (114)	225 undernourished children (200 malaria)	Recently diagnosed	NA	Microscopy	NA	NA	RDT, ELISA, PCR	168 (*Plasmodium* spp.) Severe anemia 86 (*Plasmodium* spp.)
18.	Joice et al. (2016)^34^	Malawi (1996–2011)	Case–control study	Malaria-mono-infection: 3.1–8.8 Co-infection: 1.7–3.6	Malaria-mono-infection: Male (38), Female Co-infection: Male (9), Female (11)	103 autopsy cases	NA	75 (*P. falciparum*)	IHC	Cerebral malaria 75	NA	PCR	Cerebral malaria 20/95 (*P. falciparum*)
19.	Kyeyune et al. (2014)^44^	Malawi (2002–2004)	Prospective observational study	aged < 2 years	NA	391 children with severe anemia	Recently diagnosed	183/312 (*P. falciparum*)	Microscopy	severe anemia 183	19	RDT, PCR	26/45 (*P. falciparum*) Severe anemia 26 (*P. falciparum*)
20.	Mandala et al. (2018)^35^	Malawi (2005–2006)	Cross-sectional study	1.2–4.6 years	Male (5), Female (33)	38 Cerebral malaria, 35 severe malarial anemia, control 42, HIV positive 4	Recently diagnosed	Severe malaria 59 (*P. falciparum*)	Microscopy	59 Cerebral malaria 29, Severe anemia 30	4	RDT, PCR	14 (*P. falciparum*) Cerebral malaria 9, Severe anemia 5
21.	Mouala et al. (2008)^36^	France (1996–2003)	Retrospective study	< 30, 30–39, 40–49, > 50 years	Male (99), Female (91)	190 imported malaria *P. falciparum* 178, other species 12	Undertreated	150 (*P. falciparum*)	Microscopy	Severe malaria 54	NA	PCR	28 (*P. falciparum*) Severe malaria 11 (*P. falciparum*)
22.	Munyenyembe et al. (2018)^37^	Malawi (2016–2017)	Cross-sectional study	Malaria-mono-infection: 20–67 Co-infection: 18–66	Male (50), Female (57)	107 participants with malaria	Undertreated	76 (*P. falciparum*)	Microscopy, RDT	Severe malaria 18	NA	RDT	30 (*P. falciparum*) Severe malaria 12 (*P. falciparum*)
23.	Niyongabo et al. (1994)^45^	Burundi (1991–1992)	Cross-sectional study	Adults	Male (22), Female (9)	31 cerebral malaria (*P. falciparum*)	Recently diagnosed	29 (*P. falciparum*)	Microscopy	Severe malaria 17	NA	ELISA, Western blot	Severe malaria 12 (*P. falciparum*)
24.	Nkuo-Akenji et al. (2008)^38^	Cameroon (2006)	Cross-sectional study	15–49 years: 15–25, 26–35 and 36–49 years	Male (183), Female (501)	684 outpatients	Undertreated	324 (*P. falciparum*)	Microscopy	Hyperparasitemia 84	57	RDT	201 (*P. falciparum*) Hyperparasitemia 102 (*P. falciparum*)
25.	Otieno et al. (2006)^46^	Kenya	A prospective cohort study	Children < 2 years: Malaria-mono-infection: 11.66 (0.45) Co-infection: 12.04 (1.34)	Malaria-mono-infection: Male (109), Female (85) Co-infection: Male (12), Female (12)	317 Children	Recently diagnosed	194 (*P. falciparum*)	Microscopy	Severe anemia 37	NA	RDT, PCR	23 (*P. falciparum*) Severe anemia 15 (*P. falciparum*)
26.	Sandie et al. (2019)^39^	Cameroon (2014)	Cross-sectional study	1–72 years	Male (112), Female (299)	HIV positive patients	Recently diagnosed and undertreated	34 (*Plasmodium* spp.)	Microscopy	Severe anemia 1/3	285	RDT	24 (*Plasmodium* spp.) Severe anemia 1/9 (*Plasmodium* spp.)
27.	Saracino et al. (2012)^40^	Mozambique (2010)	Cross-sectional study	> 15 years	Male (262), Female (168)	330 adult patients	Undertreated	39 (*P. falciparum*)	Microscopy, RDT	Severe malaria 17	NA	RDT	51 (*P. falciparum*) Severe malaria 29 (*P. falciparum*)
28.	Tagoe DNA and Boachie (2012)^41^	Ghana	Cross-sectional study	18–65 years	Male (59), Female (161)	220 adults with HIV	Undertreated	NA	Microscopy, RDT	NA	186	NA	34 (*P. falciparum*) Severe anemia 6 (*P. falciparum*)
29.	Tay et al. (2015)^17^	Ghana (2011–2012)	Cross-sectional study	1–73 years: 1–4 (12), 5–9 (2), 10–14 (9), 15–24 (37), 25–34 (147), 35–44 (138), 45–60 (69), > 60 (5)	Male (108), Female (292)	400 HIV sero-positive participants	Undertreated and immunosuppressed	NA	Microscopy	NA	326	RDT	47 (*Plasmodium* spp.) Severe anemia 11 (*Plasmodium* spp.)

*ELISA* enzyme-linked immunosorbent assay, *GCS* Glasgow Coma Scale, *IHC* immunohistochemistry, *NA* not applicable, *P.* *Plasmodium*, *PCR* polymerase chain reaction, *RDT* rapid diagnostic testing.

**Table 2:** Parasitemia level and leukocyte differential counts in co-infections and *Plasmodium* mono-infections.

No.	Author, year	Parasitemia level (cells/µL)		Leukocyte counts (10^3^ cells/µL)		Neutrophil counts (10^3^ cells/µL)		Lymphocyte counts (10^3^ cells/µL)	
		Co-infection	*Plasmodium* mono-infection	Co-infection	*Plasmodium* mono-infection	Co-infection	*Plasmodium* mono-infection	Co-infection	*Plasmodium* mono-infection
1.	Addissie et al. (2007)^21^	NA	NA	NA	NA	NA	NA	NA	NA
2.	Amodu-Sanni et al. (2020)^22^	NA	NA	NA	NA	NA	NA	NA	NA
3.	Berg et al. (2014)^2^	NA	NA	NA	NA	NA	NA	NA	NA
4.	Berg et al. (2008)^23^	NA	NA	NA	NA	NA	NA	NA	NA
5.	Berg et al. (2020)^24^	NA	NA	NA	NA	NA	NA	NA	NA
6.	Berkley et al. (2009)^25^	37,500 (2680–172,150) (n = 133)	22,352 (2213–142,590) (n = 938)	NA	NA	NA	NA	NA	NA
7.	Beyene et al. (2017)^26^	NA	NA	NA	NA	NA	NA	NA	NA
8.	Bronzan et al. (2007)^27^	52,048 (n = 178)	40,356 (n = 941)	10.5	11.4	NA	NA	NA	NA
9.	Chalwe et al. (2009)^28^	43,314 (25,467–81,145) (n = 27)	11,745 and 38,942 (n = 2)	6.9 (3.9)	4.2 and 8.7	NA	NA	1.73 (0.43)	1.16 (0.58)
10.	Cohen et al. (2005)^15^	NA	NA	NA	NA	NA	NA	NA	NA
11.	Davenport et al. (2010)^29^	16,220 (43,127) (n = 24)	22,281 (51,064) (n = 406)	13.3 (8.4)	11.2 (6.6)	35.6 (21.1)	41.0 (22.8)	49.4 (16.7)	50.0 (19.2)
12.	Grimwade et al. (2004)^42^	NA	NA	NA	NA	NA	NA	NA	NA
13.	Hendriksen et al. (2012)^30^	Aged < 15: 47,141 (38,005–58,474) (n = 74) Aged ≥ 15: 133,653 (59,082–302,343) (n = 581)	Aged < 15: 68,320 (37,680–123,874) (n = 49) Aged ≥ 15: 61,525 (24,628–153,704) (n = 19)	NA	NA	NA	NA	NA	NA
14.	Hochman et al. (2015)^31^	CM1: 98,300 (48,200–324,800) (n = 7) CM2: 56,400 (28,800–308,700) (n = 7)	CM1: 49,200 (5,100–717,600) (n = 5) CM2: 13,200 (7,500–433,300) (n = 33)	CM1: 14.5 (12.1–17.1) CM2: 13.2 (10.9–19.3)	CM1: 11.2 (7.3–15.7) CM2: 12.4 (9.1–21.5)	NA	NA	CM1: 2.4 (1.5, 5.1) CM2: 2.7 (1.5–5.3)	CM1: 2.3 (2–5.5) CM2: 5.3 (2.2–7.8)
15.	Huson et al. (2015)^32^	54,000 (17,340–134,700) (n = 14)	4,740 (1,300– 18,300) (n = 113)	5.1 (3.3–6.6)	5.3 (3.9–7.2)	NA	NA	NA	NA
16.	Imani et al. (2011)^43^	Cerebral malaria 240,000 (3080–583,520) (n = 9)	Cerebral malaria 21,440 (1600–133,080) (n = 91)	NA	NA	NA	NA	NA	NA
17.	Jacques et al. (2019)^33^	NA	NA	NA	NA	NA	NA	NA	NA
18.	Joice et al. (2016)^34^	20,200 (2900–32,500) (n = 20)	7400 (800–42,400) (n = 75)	13.6 (11.9–14.7)	11.2 (8.8–18.4)	NA	NA	NA	NA
19.	Kyeyune et al. (2014)^44^	NA	NA	NA	NA	NA	NA	NA	NA
20.	Mandala et al. (2018)^35^	NA	NA	10.20 (7.85–13.75) (n = 9)	11.20 (7.90–15.30) (n = 29)	6.30 (5.23–12.35)	7.40 (4.6–12.50)	1.10 (0.85–1.73)	2.01 (1.50–3.2)
21.	Mouala et al. (2008)^36^	NA	NA	NA	NA	NA	NA	NA	NA
22.	Munyenyembe et al. (2018)^37^	NA	NA	Severe malaria 6.15 (2.9–14.2) (n = 12), uncomplicated malaria 4.3 (2.4–9) (n = 18)	Severe malaria 5 (2.4–7.25) (18), uncomplicated malaria 5.6 (3.9–10.5) (n = 58)	Severe malaria 3.46 (0.9–12.4), uncomplicated malaria 2.25 (2.1–4.1)	Severe malaria 2.72 (1.1–3.74), uncomplicated malaria 3.03 (1.7–7.7)	Severe malaria 2.25 (1.1–2.8), uncomplicated malaria 1.4 (0.62–3.15)	Severe malaria 1.26 (0.6–3.3), uncomplicated malaria 1.58 (0.74–3.04)
23.	Niyongabo et al. (1994)^45^	NA	NA	NA	NA	NA	NA	NA	NA
24.	Nkuo-Akenji et al. (2008)^38^	NA	NA	NA	NA	NA	NA	NA	NA
25.	Otieno et al. (2006)^46^	NA	NA	NA	NA	NA	NA	NA	NA
26.	Sandie et al. (2019)^39^	NA	NA	NA	NA	NA	NA	NA	NA
27.	Saracino et al. (2012)^40^	1.8 ± 1.1 (n = 51)	2.3 ± 1.2 (n = 39)	7.7 ± 2.5	7.7 ± 3.7	NA	NA	NA	NA
28.	Tagoe DNA and Boachie (2012)^41^	NA	NA	NA	NA	NA	NA	NA	NA
29.	Tay et al. (2015)^17^	NA	NA	NA	NA	NA	NA	NA	NA

*NA* not applicable.

### Severe complications in patients with *Plasmodium* spp. and HIV co-infection

The total number of 1,171 severe complications were derived from 19 studies ^2,15,17,23,24,26,27,29,30,32,34,35,38–42,44,46^. The following severe complications were frequently reported in patients with *Plasmodium* spp. and HIV co-infection: severe anaemia (25.7%, 301/1171), hyperparasitaemia (15.1%, 177/1171), cerebral malaria (14.4%, 168/1171), coma (7.60%, 89/1171), convulsion (6.83%, 80/1171), and acute renal failure (6.15%, 72/1171). Among co-infected patients, ten patients who were undertreated died as reported by Berg et al.’s study (9 cases)^24^ and Huson et al. (1 case)^32^, while one patient who was immunosuppressed died as reported by Berg et al.’s study^23^. Other severe complications reported in patients co-infected with *Plasmodium* spp. and HIV are listed in Table 3.

**Table 3:** Severe complications of co-infected patients.

No.	Authors, year	Hypotension/shock	Hyperparasitemia	Severe anemia	Acute renal failure	Metabolic acidosis	Respiratory distress	Severe anemia and respiratory distress	Hypoglycemia	Cerebral malaria	Impaired consciousness/convulsion	Impaired consciousness	Coma	Convulsion	Prostration	Bleeding	Black water fever	Jaundice	Fatal
1.	Addissie et al. (2007)^21^	NS	NS	NS	NS	NS	NS	NS	NS	NS	NS	NS	NS	NS	NS	NS	NS	NS	NS
2.	Amodu-Sanni et al. (2020)^22^	NS	NS	NS	NS	NS	NS	NS	NS	NS	NS	NS	NS	NS	NS	NS	NS	NS	NS
3.	Berg et al. (2014)^2^	NS	33	15	24	NS	25	NS	8	NS	9	NS	NS	NS	NS	13	NS	17	NS
4.	Berg et al. (2008)^23^	NS	NS	NS	6	NS	NS	NS	NS	NS	NS	NS	NS	NS	NS	NS	NS	2	1
5.	Berg et al. (2020)^24^	NS	NS	NS	NS	NS	NS	NS	NS	NS	NS	NS	NS	NS	NS	9	NS	NS	9
6.	Berkley et al. (2009)^25^	NS	NS	NS	NS	NS	NS	NS	NS	NS	NS	NS	NS	NS	NS	NS	NS	NS	NS
7.	Beyene et al. (2017)^26^	NS	NS	25	NS	NS	NS	NS	NS	NS	NS	NS	NS	NS	NS	NS	NS	NS	NS
8.	Bronzan et al. (2007)^27^	NS	NS	28	NS	NS	NS	NS	NS	86	NS	NS	NS	NS	NS	NS	NS	NS	NS
9.	Chalwe et al. (2009)^28^	NS	6	5	NS	NS	NS	NS	11	NS	NS	15	NS	6	NS	NS	NS	3	NS
10.	Cohen et al. (2005)^15^	2	3	3	14	11	NS	NS	1	3	NS	NS	NS	NS	NS	NS	NS	NS	NS
11.	Davenport et al. (2010)^29^	NS	NS	14	NS	NS	NS	NS	NS	NS	NS	NS	NS	NS	NS	NS	NS	NS	NS
12.	Grimwade et al. (2004)^42^	NS	NS	14	28	NS	NS	NS	NS	NS	NS	NS	NS	NS	NS	NS	NS NS	9	NS
13.	Hendriksen et al. (2012)^30^	6	33	18	NS	38	20	18	10	NS	NS	NS	89	74	31	NS	15	9	NS
14.	Huson et al. (2015)^32^	NS	NS	NS	NS	NS	NS	NS	NS	NS	NS	NS	NS	NS	NS	NS	NS	NS	1
15.	Hochman et al. (2015)^31^	NS	NS	NS	NS	NS	NS	NS	NS	15	NS	NS	NS	NS	NS	NS	NS	NS	NS
16.	Imani et al. (2011)^43^	NS	NS	NS	NS	NS	NS	NS	NS	9	NS	NS	NS	NS	NS	NS	NS	NS	NS
17.	Jacques et al. (2019)^33^	NS	NS	86	NS	NS	NS	NS	NS	NS	NS	NS	NS	NS	NS	NS	NS	NS	NS
18.	Joice et al. (2016)^34^	NS	NS	NS	NS	NS	NS	NS	NS	20	NS	NS	NS	NS	NS	NS	NS	NS	NS
19.	Kyeyune et al. (2014)^44^	NS	NS	26	NS	NS	NS	NS	NS	26	NS	NS	NS	NS	NS	NS	NS	NS	NS
20.	Mandala et al. (2018)^35^	NS	NS	5	NS	NS	NS	NS	NS	9	NS	NS	NS	NS	NS	NS	NS	NS	NS
21.	Mouala et al. (2008)^36^	NS	NS	NS	NS	NS	NS	NS	NS	NS	NS	NS	NS	NS	NS	NS	NS	NS	NS
22.	Munyenyembe et al. (2018)^37^	NS	NS	NS	NS	NS	NS	NS	NS	NS	NS	NS	NS	NS	NS	NS	NS	NS	NS
23.	Niyongabo et al. (1994)^45^	NS	NS	NS	NS	NS	NS	NS	NS	NS	NS	NS	NS	NS	NS	NS	NS	NS	NS
24.	Nkuo-Akenji et al. (2008)^38^	NS	102	NS	NS	NS	NS	NS	NS	NS	NS	NS	NS	NS	NS	NS	NS	NS	NS
25.	Otieno et al. (2006)^46^	NS	NS	15	NS	NS	NS	NS	NS	NS	NS	NS	NS	NS	NS	NS	NS	NS	NS
26.	Sandie et al. (2019)^39^	NS	NS	1	NS	NS	NS	NS	NS	NS	NS	NS	NS	NS	NS	NS	NS	NS	NS
27.	Saracino et al. (2012)^40^	NS	NS	29	NS	NS	NS	NS	NS	NS	NS	NS	NS	NS	NS	NS	NS	NS	NS
28.	Tagoe DNA and Boachie (2012)^41^	NS	NS	6	NS	NS	NS	NS	NS	NS	NS	NS	NS	NS	NS	NS	NS	NS	NS
29.	Tay et al. (2015)^17^	NS	NS	11	NS	NS	NS	NS	NS	NS	NS	NS	NS	NS	NS	NS	NS	NS	NS
	Total complications (1171)	8	177	301	72	49	45	18	30	168	9	15	89	80	31	22	15	31	11
	Percentage	0.68	15.1	25.7	6.15	4.18	3.84	1.54	2.56	14.35	0.77	1.28	7.60	6.83	2.65	1.88	1.28	2.65	0.94

*NS* Not specified.

### Risk of bias in individual studies

Of the 29 studies included, all included studies were judged to be of high quality (≥ 7 stars). Twenty-three studies were rated with nine stars, whereas six studies^27,31,34,35,44,45^ were rated with eight stars because they did not report the information on several non-SM patients with co-infection, which was the primary outcome of the present study. Table 4 provides the data on the risk of bias of the included studies.

**Table 4:** Quality of the included studies.

No.	Reference	Selection				Compatibility	Exposure			Total score (9)	Rating (high, moderate, low quality)
		Is the case definition adequate?	Representativeness of the cases	Selection of controls	Definition of controls		Ascertainment of exposure	Same method of ascertainment for cases and controls	Non–response rate		
1.	Addissie et al. (2007)^21^	*	*	*	*	**	*	*	*	9	High
2.	Amodu-Sanni et al. (2020)^22^	*	*	*	*	**	*	*	*	9	High
3.	Berg et al. (2014)^2^	*	*	*	*	**	*	*	*	9	High
4.	Berg et al. (2008)^23^	*	*	*	*	**	*	*	*	9	High
5.	Berg et al. (2020)^24^	*	*	*	*	**	*	*	*	9	High
6.	Berkley et al. (2009)^25^	*	*	*	*	**	*	*	*	9	High
7.	Beyene et al. (2017)^26^	*	*	*	*	**	*	*	*	9	High
8.	Bronzan et al. (2007)^27^	*	*	*	*	*	*	*	*	8	High
9.	Chalwe et al. (2009)^28^	*	*	*	*	**	*	*	*	9	High
10.	Cohen et al. (2005)^15^	*	*	*	*	**	*	*	*	9	High
11.	Davenport et al. (2010)^29^	*	*	*	*	**	*	*	*	9	High
12.	Grimwade et al. (2004)^42^	*	*	*	*	**	*	*	*	9	High
13.	Hendriksen et al. (2012)^30^	*	*	*	*	**	*	*	*	9	High
14.	Hochman et al. (2015)^31^	*	*	*	*	*	*	*	*	8	High
15.	Huson et al. (2015)^32^	*	*	*	*	**	*	*	*	9	High
16.	Imani et al. (2011)^43^	*	*	*	*	**	*	*	*	9	High
17.	Jacques et al. (2019)^33^	*	*	*	*	**	*	*	*	9	High
18.	Joice et al. (2016)^34^	*	*	*	*	*	*	*	*	8	High
19.	Kyeyune et al. (2014)^44^	*	*	*	*	*	*	*	*	8	High
20.	Mandala et al. (2018)^35^	*	*	*	*	*	*	*	*	8	High
21.	Mouala et al. (2008)^36^	*	*	*	*	**	*	*	*	9	High
22.	Munyenyembe et al. (2018)^37^	*	*	*	*	**	*	*	*	9	High
23.	Niyongabo et al. (1994)^45^	*	*	*	*	*	*	*	*	8	High
24.	Nkuo-Akenji et al. (2008)^38^	*	*	*	*	**	*	*	*	9	High
25.	Otieno et al. (2006)^46^	*	*	*	*	**	*	*	*	9	High
26.	Sandie et al. (2019)^39^	*	*	*	*	**	*	*	*	9	High
27.	Saracino et al. (2012)^40^	*	*	*	*	**	*	*	*	9	High
28.	Tagoe DNA and Boachie (2012)^41^	*	*	*	*	**	*	*	*	9	High
29.	Tay et al. (2015)^17^	*	*	*	*	**	*	*	*	9	High

### Pooled prevalence of SM in patients with *Plasmodium* spp. and HIV co-infection

**Figure 2:**
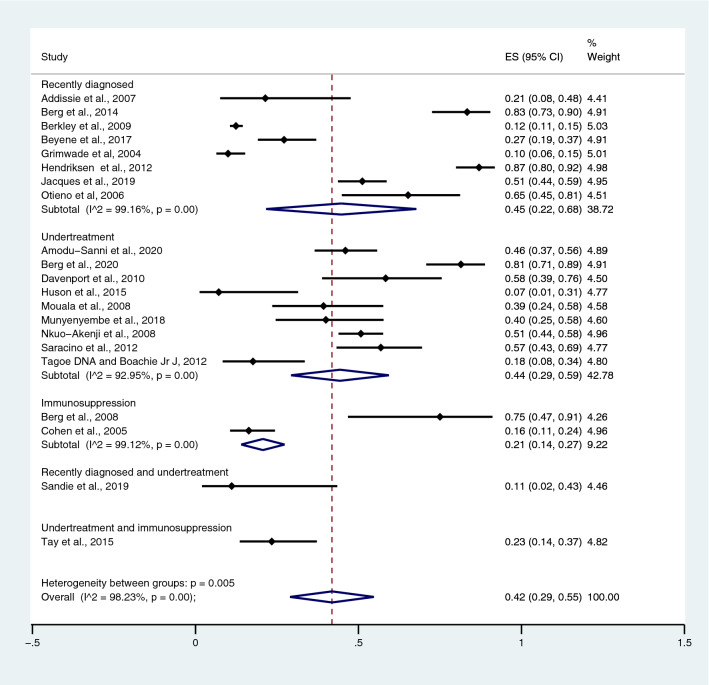
The pooled prevalence estimate of severe malaria in *Plasmodium* spp. and HIV co-infected patients.

The number of *Plasmodium* spp. and HIV co-infected patients with SM and the total number of co-infected patients were analysed to estimate the pooled prevalence of SM in patients with co-infection. Overall, the pooled prevalence of SM in patients with *Plasmodium* spp. and HIV co-infection was 42.0%, according to 21 studies (95% CI 29.0–55.0%; I^2^, 98.2%) (Fig. 2). The highest prevalence estimate (87%) was found in the study by Hendriksen et al.^30^, whereas the lowest prevalence estimate (7%) was observed in the study by Huson et al.^32^. Prevalence estimates were stratified by the time of detection of HIV infection; the prevalence of SM among co-infected patients in whom HIV had been recently diagnosed was 45.0%, according to eight studies (95% CI 22.0–68.0%; I^2^, 99.2%); among those who received undertreatment, 44.0% according to nine studies (95% CI, 29.0%–59.0%; I^2^, 93.0%) and among those who were immunosuppressed, 21.0% according to two studies (95% CI 14.0–27.0%; I^2^, 99.1%).

### The odds of SM in *Plasmodium* spp. and HIV co-infected patients

**Figure 3:**
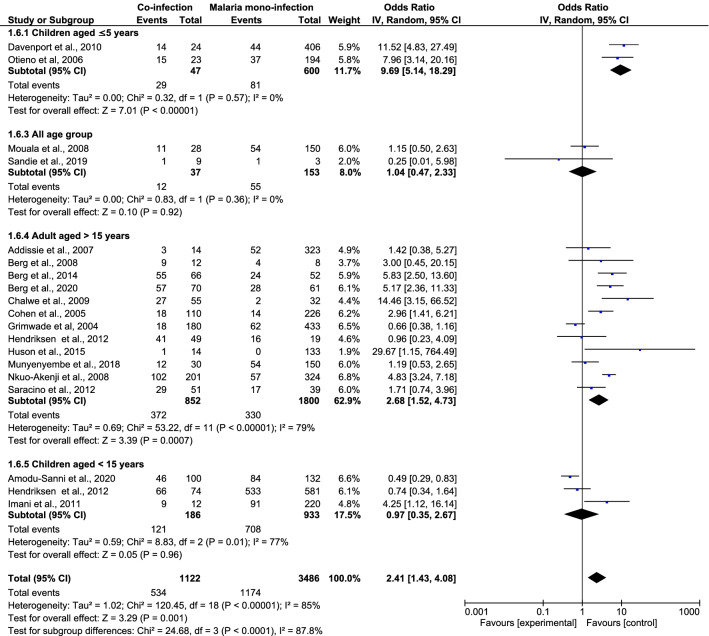
The risk of severe malaria in *Plasmodium* spp. and HIV co-infected patients.

When the number of *Plasmodium* spp. and HIV co-infected patients with SM were compared with the number of malaria mono-infected patients with SM, a significantly increased odds of SM were found in the former group, according to 19 studies (OR 2.41; 95% CI 1.43–4.08; I^2^ = 85.0%; *P* = 0.001; 19 studies) (Fig. 3). As heterogeneity was high (I^2^ statistic = 87.0%), the Random Effects model was used in the present analysis. The source of heterogeneity was identified by a subgroup analysis of the patients’ age. The subgroup analysis revealed that the odds of developing SM were significant in children aged < 5 years according to two studies (OR 9.69; 95% CI 5.14–18.3; I^2^, 0%; *P* < 0.0001) and in adults aged > 15 years who were co-infected with two pathogens according to 12 studies (OR 2.68; 95% CI 1.52–4.73; I^2^, 79.0%; *P* = 0.0007). The odds of malaria did not differ between co-infected patients and those with *Plasmodium* mono-infection in three studies that included children < 15 years of age (OR, 0.97; 95% CI, 0.35–2.67; I^2^, 77%; *P* = 0.96) or among all age groups in three studies (OR, 1.04; 95% CI, 0.47–2.33; I^2^, 0%; *P* = 0.92).

### Parasite density, leukocyte count, and differential counts

**Figure 4:**
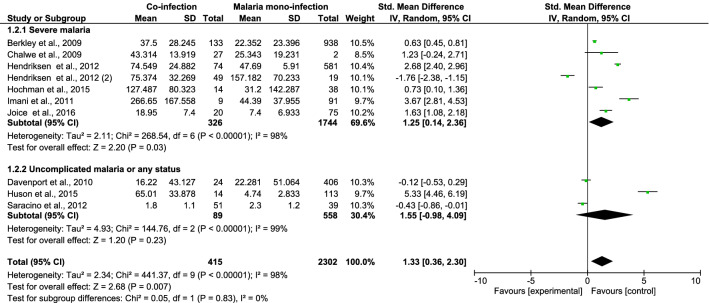
The pooled MD of parasite density between *Plasmodium* spp. and HIV co-infected and *Plasmodium* spp. patients.

**Figure 5:**
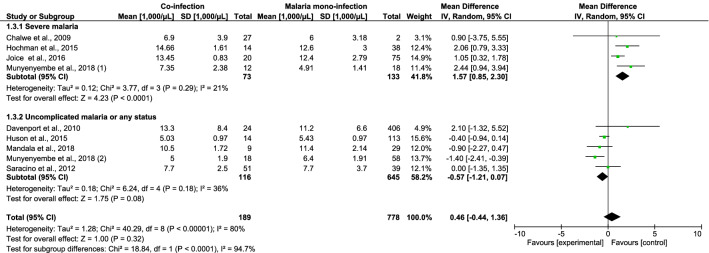
The pooled MD of total leukocyte between *Plasmodium* spp. and HIV co-infected and *Plasmodium* spp. patients.

**Figure 6:**
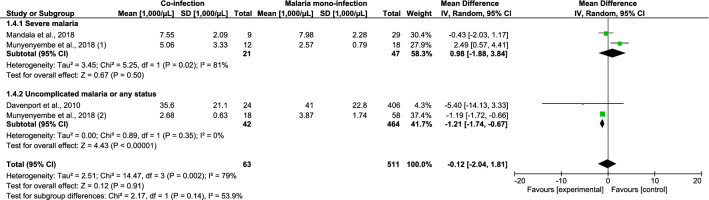
The pooled MD of neutrophil counts between *Plasmodium* spp. and HIV co-infected and *Plasmodium* spp. patients.

**Figure 7:**
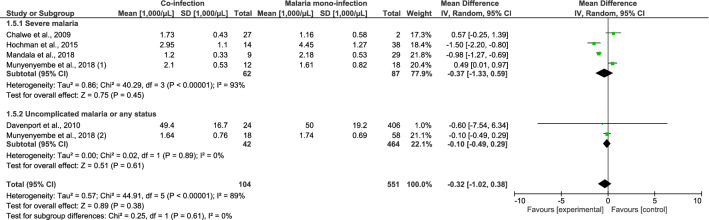
The pooled MD of lymphocyte counts between *Plasmodium* spp. and HIV co-infected and *Plasmodium* spp. patients.

The differences in parasite density, leukocyte counts, and differential counts of *Plasmodium* spp. and HIV co-infected and malaria mono-infected patients with SM were estimated. Patients co-infected with *Plasmodium* spp. and HIV who had SM had a higher mean parasite density than patients with *Plasmodium* mono-infection, according to six studies (standardised mean difference [SMD], 1.25; 95% CI 0.14–2.36; I^2^, 97%; *P* = 0.03) (Fig. 4). Co-infected patients with SM had higher leukocyte counts than patients with *Plasmodium* mono-infection, according to four studies (mean difference [MD], 1570 cells/µL; 95% CI 850–2300 cells/µL; I^2^, 21%; *P* < 0.0001) (Fig. 5). The mean neutrophil counts of patients with *Plasmodium* and HIV co-infection and SM as well as patients with *Plasmodium* mono-infection did not significantly differ according to two studies (MD, 980 cells/µL; 95% CI − 1880 to 3840 cells/µL; I^2^, 81.0%; *P* = 0.5; Fig. 6). The lymphocyte counts in *Plasmodium* spp. and HIV co-infected individuals with SM and those in *Plasmodium* mono-infected individuals were also similar according to four studies (MD, 370 cells/µL; 95% CI − 1330 to 590 cells/µL; I^2^, 93.0%; *P* = 0.45; Fig. 7).

### Publication bias

**Figure 8:**
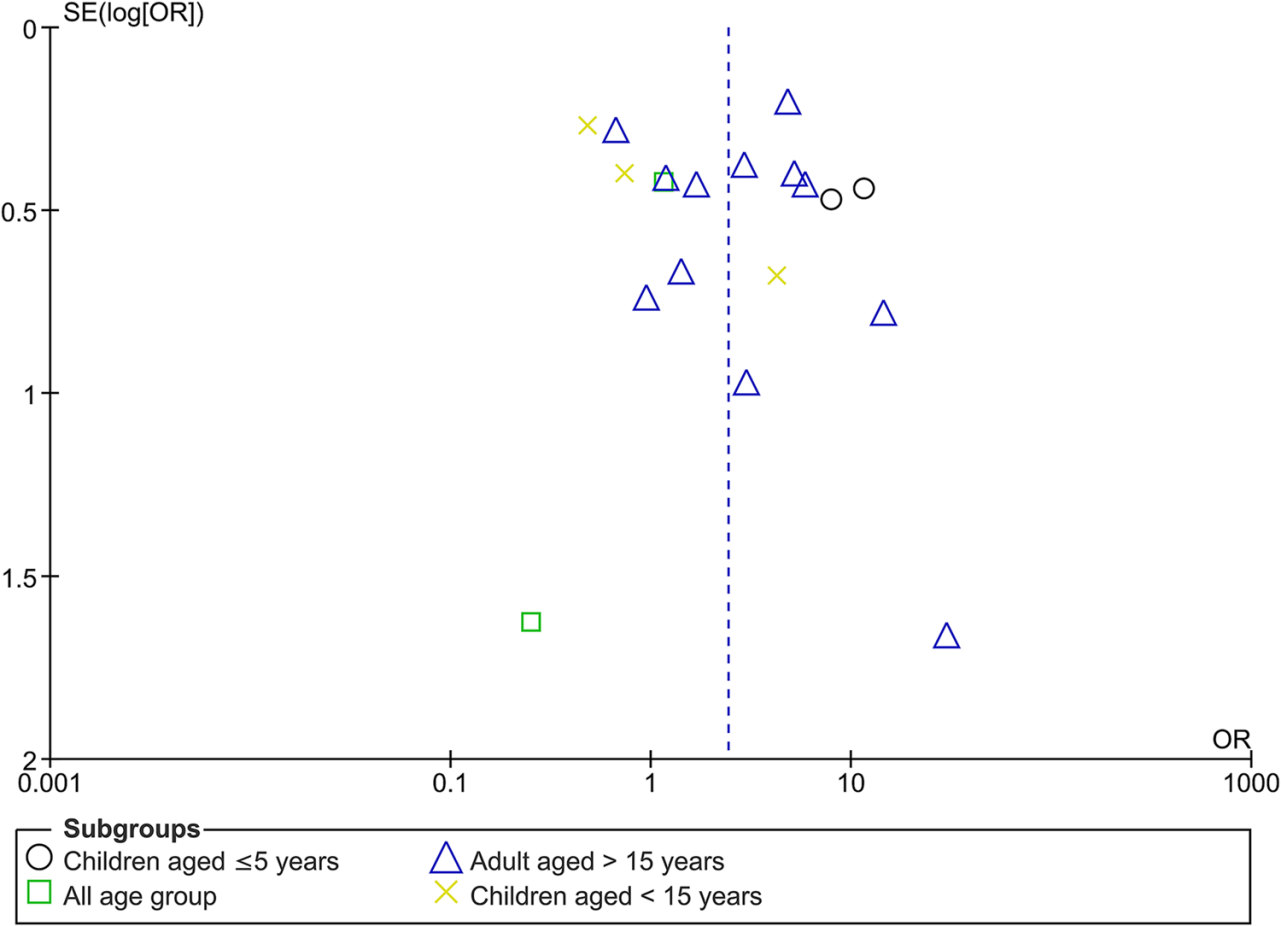
The funnel plot demonstrating publication bias across the included studies.

There was an indication of publication bias across the included studies, as demonstrated by the asymmetrical distribution of the funnel plot (Fig. 8).

### Sensitivity analysis

**Figure 9:**
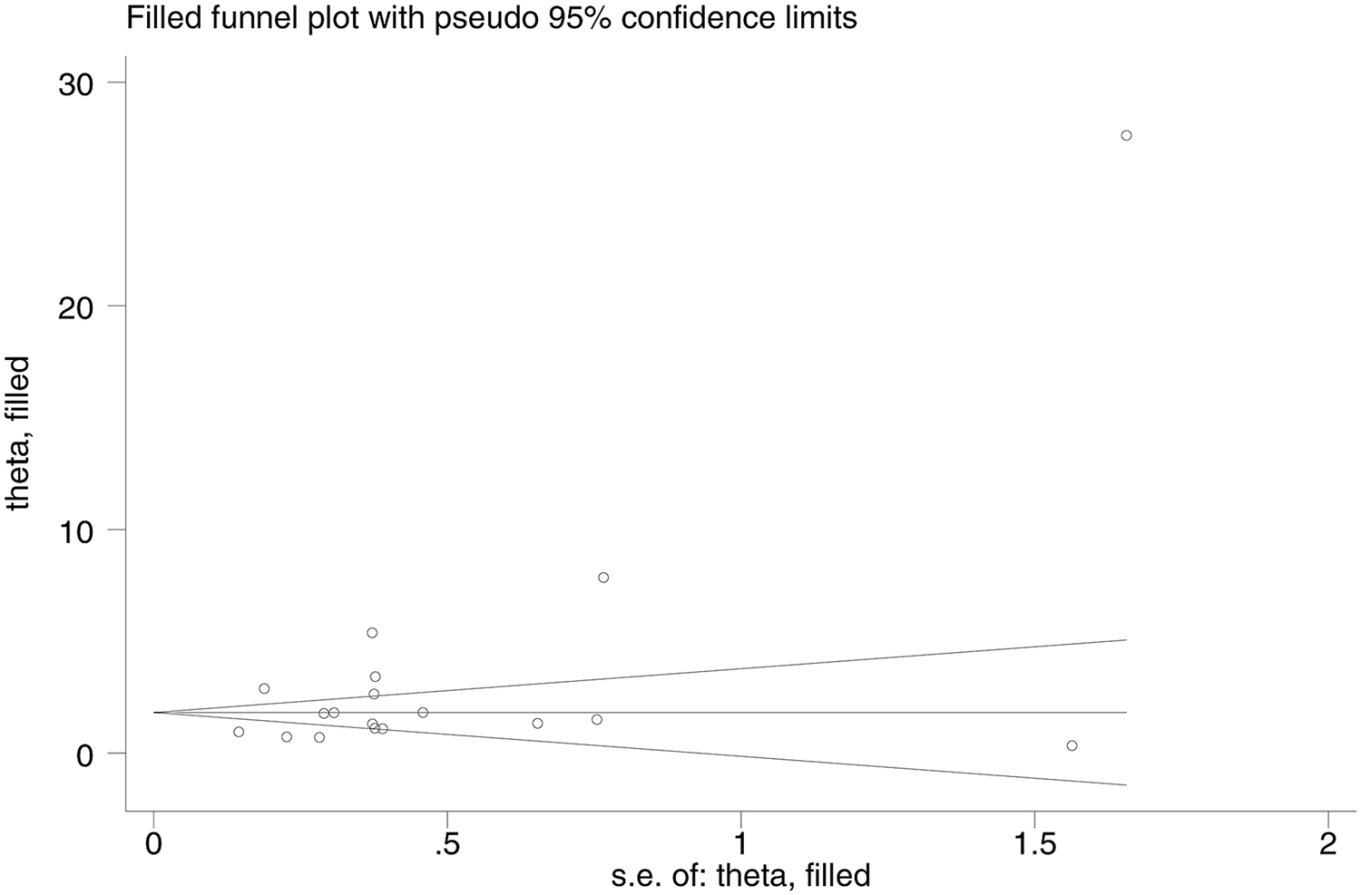
The funnel plot after trim-and-fill method was performed.

Because of the publication bias indicated in Fig. 8, we used the trim-and-fill method to evaluate the odds of SM caused by malaria and HIV co-infection in 18 studies. We found that the OR for the Fixed Effects model was 1.82 (*P* < 0.001; 95% CI 1.67–1.96), whereas the OR for the Random Effects model was 3.01 (*P* < 0.001; 95% CI 2.14–3.88; Fig. 9). We also used the trim-and-fill method to conduct the sensitivity analysis for the pooled prevalence of SM among co-infected patients. The pooled prevalence estimated by the Fixed Effects model was 13.5% (95% CI 12.2–14.8%), and that estimated by the Random Effects model was 16% (95% CI 2–29.9%).

## Discussion

Most studies reporting on co-infection with *Plasmodium* spp. and HIV were performed in Sub-Saharan Africa. The geographical overlap of these two types of infections has raised several research gaps as to how one infection influences the severity of the other. Our meta-analysis demonstrated a high prevalence of SM (43.0%) among patients with *Plasmodium* spp. and HIV co-infection, according to the data from 23 of 29 included studies. When the pooled prevalence of SM among co-infected patients was stratified by the time of detection of HIV infection, the prevalence of SM among co-infected patients recently diagnosed with HIV infection (48.0%), that among patients who were undertreated (44.0%), and that among immunosuppressed patients (45.0%) did not significantly differ, and the degree of SM severity in each subgroup was highly heterogeneous. Our sensitivity analysis of prevalence of SM among co-infected patients showed the prevalence of SM among co-infected patients were 13.5% by the Random Effects model, and 16% by the Fixed Effects model. This results suggested that the meta-analysis had the robustness of the conclusions that patients with *Plasmodium* spp. and HIV co-infection developed SM. The severity of malaria in patients with *Plasmodium* spp. and HIV co-infection may be caused by a low immune response, particularly a lower of CD4+ T cells in patients with HIV, leading to the uncontrolled number of malaria parasites, which may lead to SM^23,36,47–49^. A previous study suggested that the incidence of clinical malaria episodes was reported to be higher in patients with a CD4 cell count of < 200 cells/μL compared with those with a CD4 cell count of > 500 cells/µL^50^. Previous studies suggested that co-infection can facilitate the rate of malaria transmission by the process of CD4 cell activation, up-regulation of pro-inflammatory and cytokine production, and T-cell activation resulting in a reduction in the immune response^49,51^.

The study conducted in Mozambique demonstrated the highest prevalence of SM among children co-infected with *Plasmodium* spp. and HIV, who were characterised by undernourishment, severe acidosis, severe anaemia, respiratory distress, and elevated blood urea nitrogen concentrations^30^. The high prevalence of SM in that study might be attributable to the fact that 896 patients suspected of having SM were enrolled. Contrarily, the study with the lowest prevalence (7.0%), that of Huson et al.^32^, was a prospective observational study of 103 patients with sepsis and 127 with malaria and 60 HIV-infected individuals as a control group.

Our meta-analysis showed a significantly increased odds of SM in patients with *Plasmodium* spp. and HIV co-infection compared with those with *Plasmodium* spp. mono-infection. Our meta-analysis the odds of developing SM in patients co-infected with *Plasmodium* spp. and HIV depend on age. Although the higher odds of developing SM in adults than in children had been reported^25,27,29,47,52^, our meta-analysis demonstrated that the odds of developing SM were higher in children younger than five years and in children younger than 15 years. In addition, the odds of SM among co-infected children younger than five years (OR 9.69) were higher than those among co-infected adults older than 15 years (OR 2.68). Our sensitivity analysis of odds of SM in patients with *Plasmodium* spp. and HIV co-infection compared with those with *Plasmodium* spp. mono-infection showed the odds of SM among co-infected patients were higher than those among mono-infected patients (OR, 1.82 by the Fixed Effect model; OR, 3.0 by the Random Effects model). These results suggested that the meta-analysis had the robustness of the conclusions that patients with *Plasmodium* spp. and HIV co-infection increased odds of SM compared with those with *Plasmodium* spp. mono-infection. The development of SM among adults could be reflected by a failure to acquire immunity, which resulted in a higher parasite density among patients co-infected with *Plasmodium* spp. and HIV^47^. Conversely, *Plasmodium* spp. and HIV co-infection in children was associated with the rapid onset of cerebral malaria mediated by defects in macrophage phagocytosis^34^. This was supported by a previous study demonstrating lower absolute counts of CD4+ T cells, B cells, and NK cells in co-infected children who developed cerebral malaria^35^. That previous study demonstrated that HIV-positive patients are prone to additional opportunistic infections and febrile illnesses, which may be difficult to clinically distinguish from malaria^23^. Co-infection with *Plasmodium* spp. and HIV has been associated with a reduction in anticoagulant protein S and markers of endothelial activation, resulting in increased morbidity among co-infected patients^32^.

Our meta-analysis found that *Plasmodium* spp. and HIV co-infected patients with SM had a higher parasite density than *Plasmodium* spp. mono-infected patients with SM. We found that children younger than 5 years^25,34^ and children younger than 15 years^31,43^ who were co-infected with *Plasmodium* spp. and HIV and had SM had higher parasite densities than children with *Plasmodium* mono-infection. However, the study of adults aged 15–49 years that was conducted in Zambia^28^ demonstrated no difference in the mean parasite densities, whereas the study of both co-infected children younger than 15 years and adults older than 15 years that was conducted in Mozambique demonstrated that the SMD of parasite density was higher in children and lower in adults^30^. In patients with *Plasmodium* spp. and HIV co-infection, it was reported that malaria caused an increase in transitory HIV viral load^53^ and that HIV infection caused an increased susceptibility to malaria infection^53^ as well as induced more severe parasitaemia and higher rates of treatment failure^13^. These likely effects of HIV infection lead to impairment of the immune system, resulting in reduced control of parasite multiplication^50^.

Only a few studies have reported on the effects of co-infection on haematological parameters such as leukocytes, platelet counts, and haemoglobin levels. Our meta-analysis showed that *Plasmodium* spp. and HIV co-infected patients with SM had higher leukocyte counts than patients with *Plasmodium* spp. mono-infection. The leukocyte counts, particularly the neutrophil count, were significantly higher in patients with high parasitaemia compared with those with low and moderate parasitaemia, whereas lymphocyte counts were significantly lower in patients with high parasitaemia^54^. Our meta-analysis revealed higher leucocyte counts among studies conducted in Malawi during the periods of 1996–2011^34^, 1996–2010^31^, and 2016–2017^37^, whereas the study conducted in Zambia during 2004–2005^28^ demonstrated no differences in leucocyte counts. This difference might be explained by the fact that the study conducted in Zambia included HIV-infected patients who were immunosuppressed^28^. Although our meta-analysis demonstrated the differences in leucocyte counts, no difference in neutrophil counts or lymphocyte counts was observed. Among individual studies, the neutrophil counts were higher in the study conducted in Malawi in 2016–2017^37^ but did not differ in the study conducted in Malawi during 2005–2006^35^. Only these two studies, however, contained information on neutrophil counts. Therefore, the difference in the leucocyte counts should be investigated further.

Our meta-analysis of lymphocyte counts showed lower lymphocyte counts in two studies conducted in Malawi during the periods of 2005–2006^35^ and 1996–2010^31^ but higher lymphocyte counts in the study conducted in Malawi during 2016–2017^37^. The heterogeneity of lymphocyte counts among the three studies might be explained by the fact that two of these studies included patients who have recently been diagnosed with HIV^31,35^, and the other study included HIV-infected patients who were undertreated^37^. These findings were in agreement with that of a previous study that demonstrated that a lower lymphocyte count in HIV-infected patients was associated with a more clinically advanced disease^55^. For other haematological changes in patients with *Plasmodium* spp. and HIV co-infection, such as red blood cell parameters, another previous study demonstrated that severe anaemia was caused by a reduction in erythropoiesis^29^.

Previous studies have shown that the mortality risk among individuals with *Plasmodium* spp. and HIV co-infection was twice as high as those with HIV mono-infection^38,42,56^. The mortality caused by the *Plasmodium* spp. and HIV co-infection was reported to be 282% higher in children and 64% higher in adults with SM compared to HIV-negative patients^30^. A previous study suggested that the severity and mortality of immunosuppression by HIV might be associated with hypoglycaemia and hypotension^23^.

Our study had several limitations. First, we excluded full clinical drug trials because our objective was to investigate the odds of SM in co-infected patients who did not receive any malaria treatment. A further meta-analytic study of the risk of SM in full clinical trials should be conducted. Second, patients with HIV status who rejected malaria testing or were not tested for malaria may have resulted in the underreporting of HIV and malaria co-infection, because HIV patients may present with atypical signs and symptoms of malaria^57^. Third, the difference in the CD4 cell count between patients with co-infection and those with *Plasmodium* mono-infection could not be meta-analysed as the CD4 data reported by some included studies were insufficient. Clinicians in the regions where both *Plasmodium* spp. and HIV are endemic should carefully consider co-infection as a differential diagnosis to prevent SM. Moreover, an early evaluation of HIV patients with suspected malaria may help reduce disease severity and mortality. Further longitudinal studies should focus on the impact of HIV on malaria infection to inform the management of co-infected individuals living with HIV/AIDS. In conclusion, our systematic review and meta-analysis demonstrated that *Plasmodium* spp. and HIV co-infection could lead to SM. As patients with *Plasmodium* spp. and HIV co-infection had a greater risk of developing SM than those with *Plasmodium* spp. mono-infection, it is necessary to diagnose and treat patients with *Plasmodium* spp. and HIV co-infection to reduce the number of cases of SM and death from co-morbidities.

## Methods

### Data sources and search strategy

The present systematic review and meta-analysis adhered to the Preferred Reporting Items for Systematic Reviews and Meta-Analysis (PRISMA)^58^. The searches were performed systematically in three databases, including PubMed, Scopus, and the Web of Science. The search terms: ‘(malaria or *Plasmodium*) AND HIV AND (coinfection OR co-infection)’ were used for the searches, applying search strategies relevant to each of the individual databases. Table S1 describes the details of the search strategy for all research databases. The end date for the search was 5 May 2020. All relevant articles (no limitation in the year of publication but limited to the English language) reporting on SM in patients with *Plasmodium* spp. and HIV co-infection were screened for eligibility. The reference lists of included studies and review articles were examined for additional studies. Searches in other sources, including Google Scholar, were also performed to maximise the number of included studies.

### Study selection

The eligibility criteria for study inclusion were as follows: (1) cross-sectional studies, case–control or prospective studies reporting SM caused by *Plasmodium* spp. and HIV co-infection; (2) studies published in the English language, and (3) studies involving human samples. Any reports of a small number of cases (fewer than five), such as case reports, case series, commentaries, letters to editors, short reports, and research notes, were excluded from this study. As we aim to investigate the pooled prevalence of severe malaria in patients with malaria and HIV co-infection patients who did not receive any malaria treatment, rather than the incidence of severe malaria caused by co-infection, therefore, clinical drug trials were excluded from the present study. Two independent authors (MK and AM) screened abstract titles and evaluated the full-text articles according to the inclusion and exclusion criteria. Disagreements were resolved by requesting the third author (KUK) to reach a consensus.

### Data extraction

Two authors (AM and MK) extracted the data from the included studies. The following data were extracted: author’s name, publication year, study location, study period, study design, age range, sex, type and number of participants enrolled, the detection method for *Plasmodium* spp. and HIV, number of *Plasmodium* spp. and HIV co-infection, number of *Plasmodium* spp. mono-infections, number of cases of SM caused by *Plasmodium* spp. and HIV co-infection and *Plasmodium* spp. mono-infection. The extracted data were entered in a standardised form of an Excel spreadsheet (Microsoft Corporation, USA).

### Risk of bias in individual studies

The risk of bias of individual studies included in the present analysis was assessed independently by two authors (MK and FRM) using the Newcastle–Ottawa Scale for assessing the quality of nonrandomised studies in meta-analyses^59^. All included studies were judged based on three broad parameters, namely the selection of the study groups, the comparability of the groups, and the ascertainment of the outcome of interest^59^. A star system was developed for rating the quality of each included study with a ranging system from 1 to 9. The risk of bias was high if the study was rated < 7 stars, and the risk of bias was low if the study was rated ≥ 7 stars.

### Statistical analysis

The primary outcome of the present study was to estimate the pooled prevalence of SM among patients with *Plasmodium* spp. and HIV co-infection. The pooled prevalence of SM among patients with *Plasmodium* spp. and HIV co-infection was estimated using the Random Effects model (method of DerSimonian and Laird)^60^. The results were demonstrated as the pooled prevalence estimate and 95% confidence intervals (CIs) using a forest plot. The meta-analysis of pooled prevalence was performed using Stata version 12.1 (StataCorp LP, College Station, TX, USA). As mentioned above, the secondary aim of the present study was to determine whether *Plasmodium* spp. and HIV co-infection is associated with higher odds of SM when compared with *Plasmodium* spp. mono-infection. The pooled odds ratio (OR) and 95% CI was estimated using (1) the number of patients with SM in the presence of *Plasmodium* spp. and HIV co-infection and those with *Plasmodium* spp. mono-infection; (2) the total number of patients with *Plasmodium* spp. and HIV co-infection and those with *Plasmodium* spp. mono-infections. The pooled mean differences (MDs) and 95% CI between laboratory parameters, including parasite density, and leukocyte and differential counts were estimated based on the means and standard deviations (SDs) between the two groups. Medians and ranges/interquartile ranges reported by included studies were transformed to means and SDs as described elsewhere^61^. Meta-analyses of the pooled ORs and MDs were performed using Review Manager (RevMan) 5.3 software (Version 5.3, London, UK). The heterogeneity among included studies was tested and quantified by the Cochrane chi-square, and I^2^ statistics were presented in the forest plots. If the I^2^ statistic was higher than 50%, indicating substantial heterogeneity^62^, the Random Effects model was used in the meta-analysis. A subgroup analysis of age groups and locations of participants was also performed to identify any difference in the odds of SM among subgroups.

### Publication bias

Publication bias was evaluated by visual inspection of funnel plot asymmetry. Generally, if symmetry is observed, this indicates no publication bias, whereas asymmetry suggests publication bias across the included studies. If the results indicated a publication bias, we revised the estimate of the prevalence and the odds ratio after correcting for such publication bias in the sensitivity analysis using the trim-and-fill method^26^ utilizing Stata ver. 14 (Stata Corporation, College Station, TX, USA).

## Supplementary Information

Supplementary Table S1.
