# Supplementary material for: A meta-analysis on the prevalence and characteristics of severe malaria in patients with Plasmodium spp. and HIV co-infection
Source: Sci Rep. 2021 Aug 17;11:16655. doi: 10.1038/s41598-021-95591-6 (PMC8371128; doi:10.1038/s41598-021-95591-6)
Supplement: Supplementary file 1 — Supplementary Table S1. [file 41598_2021_95591_MOESM1_ESM.docx]

**A meta-analysis on the prevalence and characteristics of severe malaria among patients with *Plasmodium* spp. and HIV co-infection**

Aongart Mahittikorn^1^, Kwuntida Uthaisar Kotepui^2^, Giovanni De Jesus Milanez^3^, Frederick Ramirez Masangkay^3^, Manas Kotepui^2*^

^1^Department of Protozoology, Faculty of Tropical Medicine, Mahidol University, Bangkok, Thailand

^2^Medical Technology, School of Allied Health Sciences, Walailak University, Thasala, Nakhon Si Thammarat, Thailand

^2^Department of Medical Technology, Institute of Arts and Sciences, Far Eastern University-Manila, Manila, Philippines

**Table S1 Search term**

| **Databases** | **Search terms** | **Date** |
| --- | --- | --- |
| PubMed | (malaria or Plasmodium) AND HIV AND (coinfection OR co-infection) | 5 May 2020 |
| Scopus | (malaria or Plasmodium) AND HIV AND (coinfection OR co-infection)  Search option: All fields | 5 May 2020 |
| ISI Web of Science | (malaria or Plasmodium) AND HIV AND (coinfection OR co-infection)  Search option: All fields | 5 May 2020 |
